# Music mnemonics aid Verbal Memory and Induce Learning – Related Brain Plasticity in Multiple Sclerosis

**DOI:** 10.3389/fnhum.2014.00395

**Published:** 2014-06-13

**Authors:** Michael H. Thaut, David A. Peterson, Gerald C. McIntosh, Volker Hoemberg

**Affiliations:** ^1^Center for Biomedical Research in Music, Colorado State University, Fort Collins, CO, USA; ^2^Computational Neurobiology Laboratory, Salk Institute for Biological Studies, La Jolla, CA, USA; ^3^Institute for Neural Computation, University of California San Diego, La Jolla, CA, USA; ^4^Department of Neurology, University of Colorado Health, Fort Collins, CO, USA; ^5^Department of Neurology, SRH Rehabilitation Hospital Bad Wimpfen, Bad Wimpfen, Germany

**Keywords:** verbal memory, musical mnemonic, deep encoding, electroencephalogram, alpha/beta oscillations, learning-related neural synchrony

## Abstract

Recent research on music and brain function has suggested that the temporal pattern structure in music and rhythm can enhance cognitive functions. To further elucidate this question specifically for memory, we investigated if a musical template can enhance verbal learning in patients with multiple sclerosis (MS) and if music-assisted learning will also influence short-term, system-level brain plasticity. We measured systems-level brain activity with oscillatory network synchronization during music-assisted learning. Specifically, we measured the spectral power of 128-channel electroencephalogram (EEG) in alpha and beta frequency bands in 54 patients with MS. The study sample was randomly divided into two groups, either hearing a spoken or a musical (sung) presentation of Rey’s auditory verbal learning test. We defined the “learning-related synchronization” (LRS) as the percent change in EEG spectral power from the first time the word was presented to the average of the subsequent word encoding trials. LRS differed significantly between the music and the spoken conditions in low alpha and upper beta bands. Patients in the music condition showed overall better word memory and better word order memory and stronger bilateral frontal alpha LRS than patients in the spoken condition. The evidence suggests that a musical mnemonic recruits stronger oscillatory network synchronization in prefrontal areas in MS patients during word learning. It is suggested that the temporal structure implicit in musical stimuli enhances “deep encoding” during verbal learning and sharpens the timing of neural dynamics in brain networks degraded by demyelination in MS.

## Introduction

The past two decades have seen an increasing awareness of cognitive deficits in multiple sclerosis (MS). Many MS patients have cognitive deficits (Borghi et al., 2013; Rahn et al., 2012; Rogers and Panegyres, 2007; Amato et al., 2001; Gaudino et al., 2001; Kujala et al., 1996; Rao, 1990; Peyser et al., 1980). It is estimated that up to 65% of persons with MS suffer from cognitive impairment affecting their quality of life, vocational ability, and social function. Although cognitive impairments in MS were described already in the nineteenth century, not until 2001 standard tests were codified to measure cognitive function in MS (Rahn et al., 2012).

Memory is one of the most prevalent types of cognitive deficit in MS and some memory deficits present in early phases of the disease (Gaudino et al., 2001; Landro et al., 2000; Thornton and Raez, 1997; Kujala et al., 1996; DeLuca et al., 1994; Peyser et al., 1980). However, no convincing evidence for effective pharmacological or other treatments for memory impairment in MS does exist (He et al., 2011). Furthermore, despite new memory test development (Camp et al., 2001) and several research studies aimed at isolating the memory problems in MS (Marie and Defer, 2001; Minden et al., 1990), the exact nature of memory deficits in MS remains unclear. One major theoretical approach attributes memory deficits in MS to inadequate learning processes (DeLuca et al., 1994) including reduced information processing speed which may prevent “deep encoding” of learning material (Rao et al., 1989; Litvan et al., 1988). More important clinically, however, as Rao (1995) has ardently noted for many years, is the lack of treatments for memory dysfunction in MS (Bennett et al., 1991).

In the past two decades, research has discovered the effectiveness of music as a temporal auditory language in neurorehabilitation (Thaut, 2005). The initial discoveries established the effect of music and rhythm in motor therapies, most comprehensively in stroke and Parkinson’s disease (DeDreu et al., 2012; Thaut et al., 2007; Thaut et al., 1997; Thaut et al., 1996). Physiological priming (Rossignol and Melvill Jones, 1976), anticipatory perceptual cue timing, and neural auditory motor entrainment (Grahn and Watson, 2013; Thaut, 2013) have been proposed among the most prevalent underlying mechanisms. Music and rhythm intervention techniques (c.f., Rhythmic Auditory Stimulation RAS) are now considered evidence based and are widely used within neurologic rehabilitation (Hoemberg, 2013).

However, the recognition that timing and sequencing also have a critical function in cognitive abilities (Conway et al., 2009) has led to research investigating the potential role of music and rhythm as cognitive rehabilitation technique. Sound in music is inherently temporal and sequential and may serve as a “scaffold” to bootstrap the representation of temporal sequential patterns in cognitive functions such as memory (Conway et al., 2009). In support of this concept, many clinical reports have emphasized the relative “survival” of musical memories in neurologic memory disorders (Haslam and Cook, 2002; Baur et al., 2000), dementia, and Alzheimer’s disease (Son et al., 2002; Foster and Valentine, 2001). Music processing may recruit not only declarative but also more automatic, procedural learning and memory systems that are spared in amnesia.

There is considerable evidence that music can also enhance memory for non-musical material (Thaut, 2005; Ho et al., 2003; Jakobson et al., 2003; Rainey and Larsen, 2002; Wallace, 1994). Previous evidence has shown that music memory provides access to verbal knowledge in patients with memory disorders (Cavaco et al., 2012; Moussard, 2012; Simmons-Stern et al., 2012; Sarkamo et al., 2008; Mammarella et al., 2007; Haslam and Cook, 2002; Foster and Valentine, 2001; Baur et al., 2000). Specific benefits of musical mnemonic rehearsal over verbal rehearsal when learning non-musical material have been shown with learning disabled and developmentally disabled students (Kern et al., 2007; Claussen and Thaut, 1997; Wolfe and Hom, 1993; Gfeller, 1983). In a study with autistic children – however without matched controls or a comparable control condition – a structured music listening protocol has shown to enhance a broad range of cognitive functions including memory (Bettison, 1996). However, Maeller (1996) demonstrated that music can improve memory in MS patients, with a trend toward greater improvement associated with severity of the disease.

The rhythmic-melodic pattern of a song as an auditory “scaffold” to bootstrap non-musical information may offer several advantages to facilitate “deep encoding” for the learning and retrieval process (Wallace, 1994). The rhythmic and melodic structure provides a temporal cue for the temporal order and sequencing of information. Additionally, the melody provides pitch contour cues to which information units can be mapped. The phrase structure of a melody segments information into chunks or single overarching units of information with distinct sound shapes, which is especially important when information units such as words in word lists are unrelated to each other (Snyder, 2000; Deutsch, 1982). In such process, several information units become segmented into one learning unit. Chunking is an important memory strategy because it reduces memory load (Gobet et al., 2001). Another critical element for learning via musical mnemonics is the fact that musical mnemonics, such as short songs, are composed of a relatively small “alphabet” of tones/pitches to which information units from a larger “alphabet” can be mapped (Dowling, 1973). Information such as a diatonic pitch scale (seven scale tones) is much easier to group and encode than data from large alphabets and we are much more likely to retain information from several small alphabets than the same total amount of information from a large alphabet. The English language uses 26 letters and up to 40 separate phonemes. Finally, in a well composed musical mnemonic the small tonal alphabet is organized into redundant repetitive and anticipatory units which are easy to remember (Snyder, 2000). By pairing verbal material with a simple melody (e.g., one word mapped to one note), a line of several unrelated words or numbers can now be bounded and encoded into a single “small alphabet” segment (Hitch et al., 1996; Deutsch, 1982).

Research on the neural basis of music and memory has mostly focused on musical memory formation. There are some studies that have investigated neural correlates of non-musical autobiographical recall elicited by music (Ford et al., 2011; Janata, 2009). However – despite the emerging behavioral evidence for musical mnemonics to assist in non-musical learning – no research has directly studied so far the neural correlates of non-musical memory training with musical mnemonics.

In other areas of brain and behavior function, there is emerging evidence that music modulates brain activity associated with non-musical functions of the nervous system. For example, we have evidence that rhythmic entrainment can be used for sensorimotor rehabilitation: the temporal structure of music can be harnessed to rehabilitate motor function and facilitate motor plasticity in brain damaged patient populations (Rojo et al., 2011; Luft et al., 2004; Hummelsheim, 1999). As another example, musical training evokes brain plasticity during speech perception (Kraus and Chandrasekaran, 2010). Therefore, in our study we included an investigation of neural correlates related to brain plasticity during verbal memory training using musical mnemonics. Plasticity in the functional organization of brain networks is important in recovering verbal learning and memory function after, for example, traumatic brain injury (Ricker et al., 2001). Music can play a role in brain plasticity, through both its pitch characteristics (Shahin et al., 2003) and temporal structure (Pantev et al., 1999; Merzenich et al., 1993). Music training (to discriminate pitch) produces enhanced plasticity of the N1 and P2 components of the auditory evoked potential (AEP) (Shahin et al., 2003). The temporal structure of music influences the brain oscillations associated with short-term memory for auditory patterns (Peterson and Thaut, 2002).

In this study, we investigated for the first time in persons with MS whether a musical template for verbal learning not only improves learning and memory but also involves a different pattern of short-term, system-level brain plasticity measured as changes in oscillatory network synchronization. Given the practical significance of sequencing in verbal information, we specifically investigated whether music would improve learning and memory for ordered word lists. We used the spectral power of the electroencephalogram (EEG) to measure oscillatory synchronization in patients with MS while they performed Rey’s auditory verbal learning test (RAVLT).

## Materials and Methods

### Subjects

Subjects were 54 right-handed volunteers (38 female/16 male) with relapsing-remitting MS, with normal hearing, and no history of other neurological or psychiatric conditions. Subjects showed at least five brain lesions identified via MRI analysis. Subjects were stable on their immunomodulatory therapy and had <2 exacerbations within the 12-month prior to the study. Participants were not in an active exacerbation phase at the time of testing and were not treated with pulse-cortical steroids or cognition enhancing AChE inhibitors.

All subjects volunteered and provided written, informed consent approved by the institutional review board. Subjects were randomly assigned by computerized random number generator in concealed allocation to one of the two conditions: with and without a musical template, hereafter referred to as the “spoken” and “sung” conditions. Experimenters were blinded to assigned condition. There were no statistically significant differences between participant characteristics in the spoken and sung condition (Table 1).

**Table 1:** **Demographic data of MS participants: means (standard deviations)**.

	Spoken	Sung	*t*(52)	*P*
Age (years)	53.3 (9.3)	50.3 (10.1)	0.98	n.s.
Education (years)	15.3 (2.6)	14.7 (2.0)	1.15	n.s.
Private music training (years)	8.9 (13.9)	5.2 (4.5)	0.72	n.s.
EDSS	4.3 (0.9)	4.9 (1.3)	1.53	n.s.

*EDSS, extended disability status scale (Kurtzke, 1983) (0 = no neurologic impairment, 10 = death due to MS)*.

### Task and stimuli

**Figure 1:**
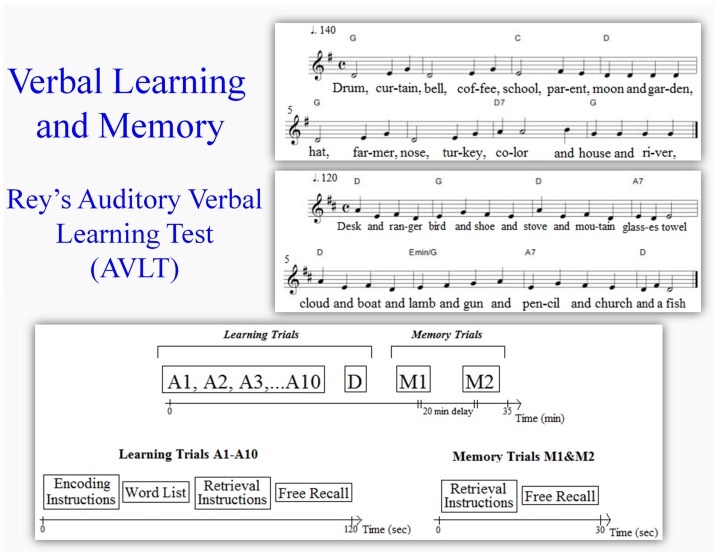
**Rey’s auditory verbal learning test (RAVLT)**. There were 10 presentations of the same word list and one presentation of a new distractor word list. On each trial, subjects hear the full list of 15 words before being prompted to recall. Subjects are asked to perform free recall of the words again (without additional presentation) in two memory tests, M1 = immediate recall after the distractor list and M2 = recall after a 20 min delay and distractor task (D, distractor task).

Unlike the more typical in-person administration of the RAVLT (Lezak, 1995), we used pre-recorded sound files and remotely recorded voice responses with the subjects isolated in a sound-proof booth to eliminate experimenter bias and to control for stimulus equality across subjects, to maximize consistency of the test procedures and to avoid the increased risk of artifacts in the simultaneously recorded EEG during interaction with another person. A single standard list of 15 words was repeated over 10 trials. Subjects were asked to recall as many words as possible after each list presentation (see Figure 1). The 15 words in the list were semantically unrelated. The listed words were presented at a rate of one per second and in the same order on every trial. On each trial, subjects were instructed to listen carefully as they would subsequently be asked to recall as many words as possible. Recall of the list (M1) was tested without further presentation of the original list after subjects heard and free-recalled a distractor list and again after a 20-min non-verbal distractor task (M2). Subjects were not given feedback on any trials.

In both conditions they were additionally instructed to recall items in the order they were presented on the word list.

Identical word lists were used in both experimental conditions. Overall durations for word list presentation in the spoken or sung conditions had the same length. List was presented via free field at 80 dB SPL. The same female voice was used to produce the sung and spoken word lists. In the musical mnemonic condition, the words were sung to the melody of an originally composed song. One-syllable words were assigned one-quarter note of 1 s duration, while two-syllable words were assigned one-eighth note of 0.5 s (500 ms) per syllable to generate melodic-rhythmic phrasing and to keep both conditions at 15 s durations (with 1 s rest at the end). We used an originally composed melody that was not familiar but was simple and repetitive in structure (AABA form).

The behavioral analysis measured results for overall recall of the words and for word chunks recalled in proper sequence, assessing chunk lengths of correct words’ pairs across the list sequence.

### Electrophysiological recording and analysis

We recorded from 128 scalp electrodes using Electrical Geodesic’s Sensor Net. Continuous EEG was recorded using low- and high-cutoff frequencies of 1 and 100 Hz, respectively, with a 1-kHz sampling frequency. We used EGI’s ocular artifact toolbox (precursor to the current Polygraphic Recording Analyzer, PRANA) to remove trials with ocular artifacts including eye movements and blinks. Spectral power was calculated over the 250–750 ms post-onset time window, common to most studies of verbal encoding (see, e.g., Staresina et al., 2005). Spectral power was computed for alpha and beta frequency bands, separately for each of the following subbands: alpha_1 (7–9 Hz), alpha_2 (9–11 Hz), alpha_3 (11–13 Hz), beta_1 (13–20 Hz), and beta_2 (20–34 Hz). Topographic EEG analysis was organized by quadrants defined by the following electrodes:

left anterior: F3, F7, FC3, FT7
right anterior: F4, F8, FC4, FT8
left posterior: CP3, TP7, P3, P7
right posterior: CP4, TP8, P4, P8.

We defined the “learning-related synchronization” (LRS) as the percent change in EEG spectral power averaged over the second through tenth encoding trials (collectively) relative to the power in the first encoding trial:

$$\text{LRS}=\frac{\text{power\_learned}}{\text{power\_notlearned}}\times 100-100.$$

Thus, LRS was defined in a fashion analogous to the more general event-related de-synchronization (ERD) or, more precisely, its inverted counterpart, the even-related synchronization (ERS) (Pfurtscheller, 1999). The primary difference is that the baseline for the LRS was not a period of time immediately preceding the trial of interest, but the first trial in which the same word was presented. By using the power ratio, we also mitigated absolute differences in subjects’ baseline spectral EEG power. The LRS was calculated individually for every subject, frequency band, and electrode. We evaluated the average LRS from each of four quadrants: left anterior, right anterior, left posterior, and right posterior. For each frequency band the quadrant LRS averaged across subjects in each condition was compared between groups to test for change in short-term, systems-level plasticity associated with learning.

## Results

In overall recall, the music condition produced statistically significant results higher than in the spoken condition [two-way ANOVA: *F* (1.52) = 4.12; *p* = 0.45; mean squared error 0.057] (Table 2).

**Table 2:** **Percentage means of recalled words at M1 and M2**.

	M1	M2
Sung	77	76.6
Spoken	64.7	59.8

**Figure 2:**
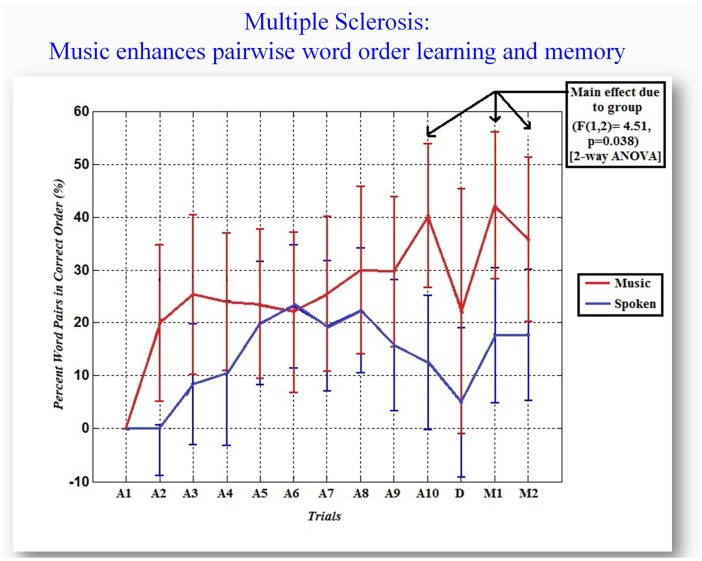
**Music enhances pair-wise word order learning and memory**. *X*-axis: trial (learning trials 1–10, distractor trial “Dd,” and memory trials 1 and 2). The % of recalled word pairs in correct order (*Y*-axis) is the change relative to trial 1. Bars are standard error.

The analysis of pair-wise word order learning showed a statistically significant advantage for recall in music than spoken learning at the end of the last learning trial and the two subsequent memory trials [*F*(1, 2) = 4.51, *p* = 0.038, two-way ANOVA] (Figure 2). Musical verbal learning induced greater increase in word order recall in early and late phases of learning, whereas spoken verbal learning induced greatest increase in word order recall during the middle phase of learning. The spoken verbal learners’ performance actually decreased slightly in the last two learning trials, and remained relatively lower than the musical verbal learners in the later recall trials.

**Figure 3:**
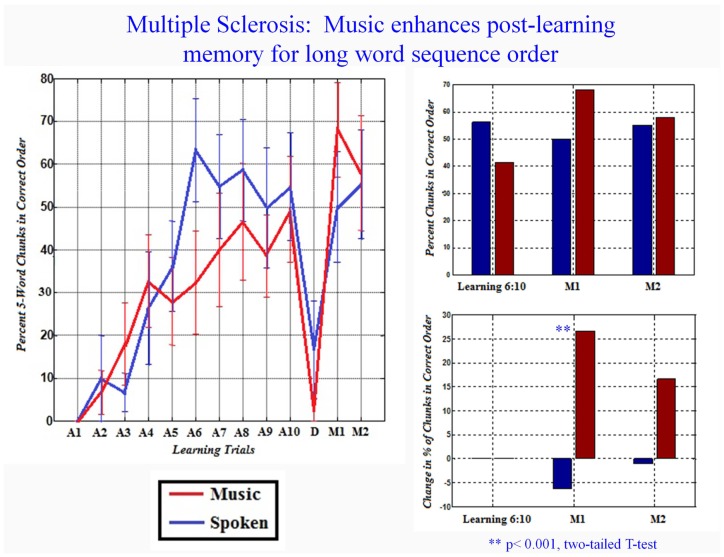
**Music enhances memory for correct five word order sequences only during recall but not during acquisition**. *X*-axis: trial (learning trial 1–10, distractor trial “D,” and memory trials 1 and 2). The % of recalled word sequences in correct order (*Y*-axis) is the change relative to trial 1. Bars are standard error. Bar graphs on right show % of correct word order for learning trials 6–10.

When analyzed for a longer word order sequence (five words in correct order), the significant advantage for music disappeared during the acquisition trial. However, during M1 the significant difference between musical and spoken condition reemerged: the change between acquisition trials 6–10 and first recall was significantly higher for music than the spoken condition (Figure 3).

**Figure 4:**
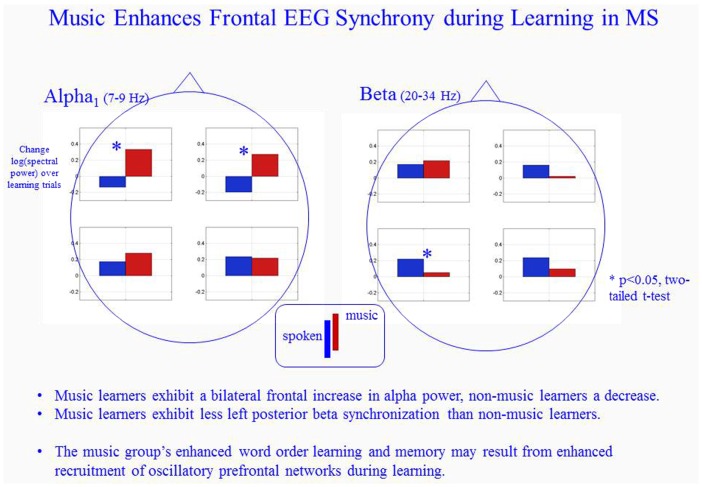
**Music induces a different form of learning-related synchronization (LRS) during verbal learning in MS than non-musical learning**. Left: music enhances bilateral frontal alpha_1_ LRS. Right: music is associated with significantly reduced left posterior beta LRS than conventional spoken verbal learning (*denotes statistically significant difference between the groups).

The stronger word order memory performance for the music condition was associated with low-alpha band (“alpha_1”) LRS in bilateral frontal areas, whereas low-alpha power actually decreased in those same areas over the course of learning in the spoken condition (Figure 4). The difference between the groups’ bilateral frontal alpha LRS was significant at the 0.05 level [*t*(14) > 2.4]. Both groups exhibited low-alpha LRS bilaterally in posterior regions, with no significant difference between the groups. In the upper beta band, both groups exhibited LRS (synchronization associated with verbal learning). In all but the left anterior quadrant, the spoken condition involved greater synchronization than the music condition, a difference that reached statistical significance in the left posterior area [*t*(14) > 2.3, *p* < 0.05].

Regression analysis between correct word order recall as a predictor of magnitude of overall word recall was highly significant in the sung condition for M1 (*p* = 0.15; *R*^2^ = 0.29; beta = −0.54; Corr. = −0.54). Furthermore, higher EDSS scores were significant predictors for word order recall, only evident in the sung condition, however for both M1 and M2 (M1: *p* = 0.001; *R*^2^ = 0.57; beta = 0.753; Corr. = 0.41) (M2: *p* = 0.005; *R*^2^ = 0.36: beta + 0.60; Corr. = −0.42).

## Discussion

Subjects in the music condition showed significantly better total word memory, and more specifically, also paired word order memory than subjects in the spoken condition. Interestingly, general performance in word memory was significantly predicted in the music condition by correct word order recall, emphasizing the critical role of temporal structure in learning and retrieval of verbal information. Subjects who had better word order memory showed better overall word recall. This finding supports notions that music’s facilitating mechanism is driven by its temporal structure sequencing and chunking unrelated information units into single segments (Beatty and Monson, 1991). Temporal structure for sequencing and a small “alphabet” scaffold to reduce memory load are – based on the study data – suggested to facilitate “deep encoding” for patients with MS during the memory task. The advantages for the music assisted over the spoken memory condition were evident consistently during the learning as well as during the final retrieval stages of the RAVLT.

Important to note, however, is the contrast in the longer word order analysis where significant advantages for music in more complex task measure only appeared in the retrieval phase and not during the actual learning trials. During the acquisition trials both conditions showed equal performance. However, the retrieval advantage for music may indicate that already during the acquisition trials the musical mnemonic facilitated deeper encoding traces for word order memory.

Theoretical approaches have assigned memory impairments in MS to inadequate learning strategies rather than insufficient retrieval abilities. However, other suggestions have been made pointing at impaired retrieval processes (Rahn et al., 2012). In either case, our data suggest that musical mnemonics may successfully operate in both memory processes by enhancing deep encoding during initial information acquisition and providing critical cues for successful memory retrieval. Our results show that the advantage for deep encoding via music during more complex measures (repeating five words in correct order vs. word pairs) may not show immediately during acquisition trials. Evidence for enhanced deep encoding may not show until the retrieval phase indicating learning strategies based on “silent” processes whose success may not become evident until recall.

Several subjects in the sung condition sang the word lists back. The last point may be of clinical importance. In an earlier study (Peterson and Thaut, 2007), we found no differences when healthy subjects learned a version of the RAVLT in sung or spoken presentation. However, they were asked to speak the word lists back in both conditions during the two memory trials. We proposed as an explanation that the incongruence between learning and recall (speak back information presented in a song) created a transfer challenge that nullified a potential benefit for music. In the current study, we therefore did not require a change in recall modality (singing vs. speaking). This adjustment resulted in a significant advantage for music-assisted learning and may be an important clinical strategy for the use of musical mnemonics training as it is codified in the Neurologic Music Therapy Taxonomy (MMT) (Thaut and Hoemberg, 2014).

One important limitation in the current study is the absence of a healthy control group to confirm the differences found in the group with MS. However, memory is considered one of the core affected cognitive functions in MS and core functions are more affected than other more peripheral cognitive functions (Calabrese and Penner, 2007). Many assessment approaches to test for memory in MS use immediate and delayed recall of word lists (Wallin et al., 2006). As such we may consider the improvements during music in learning and recall using the tasks of the RAVLT a salient contribution to cognitive treatment for patients with MS.

Subjects in the music condition showed higher low-alpha power in bilateral frontal areas than subjects in the spoken condition. The increased power is usually attributed to an increase in oscillatory synchronization in recurrent cortical networks. However, it remains unknown whether it arises from a larger population of neurons recruited into the oscillatory assemblies, or greater phase synchrony in an assembly of a given size, or some combination of both.

The remarkable increase in neuronal synchronization in cortical networks in our cohort of people with MS after music-assisted training needs to be further investigated in light of the fact that the demyelination process of the disease will affect and interrupt network dynamics of neuronal cell assemblies. One might expect heterogeneous demyelination processes to variably increase propagation delays, thereby increasing noise in phase relationships among neurons in a given cell assembly and reducing synchrony (Calabrese et al., 2009). However, in light of research showing that increased frontal alpha power may represent less brain activity (Laufs et al., 2003) and increased power has been proposed as a sign for less mental effort (Jausovec, 1998), the results may indicate that music template assisted learning resulted in less mental effort by facilitating deep encoding.

This result is further underlined by the behavioral results that higher EDSS scores (higher disease states) were correlated with higher improvements in word order recall, suggesting that patients in more severe disease stages benefited particularly from music-facilitated “deep encoding” learning strategies. The results provide evidence that melodic-rhythmic templates, as commonly inherent in the temporal structure in music, may drive internal rhythm formation in recurrent cortical networks involved in learning and memory. It is particularly noteworthy that we found different short-term plasticity between the two conditions during encoding, a phase of memory processing associated with deficits in MS (Sweet et al., 2004; Marie and Defer, 2001). At a functional level, in light of past research implicating low-alpha band power in attentional processes (Klimesch, 1999; Fuster, 1997; Klimesch, 1997), the lower alpha band results in our study suggests that the influence of the musical condition may be at least partly mediated by an influence on attention. Relatedly, our low-alpha findings may also reflect some sort of adaptive processes, as seen in a conversion from relative de-synchronization (ERD) to synchronization (ERS) as subjects’ transition from the first to subsequent minutes of listening to music (Krause et al., 1999). Future studies are needed to carefully control these various factors of modality, attention, and adaptation.

While the exact nature of the neurophysiologic mechanisms underlying the plasticity we measured with scalp EEG remains elusive, and the short-term nature of the learning task preclude changes in network structure, a few theories may be put forth. Musical mnemonics may activate or access alternative or compensatory pathway circuitry for memory functions to compensate for compromised prefrontal functions associated with learning and recall. The temporal pattern structure of a musical mnemonic may also facilitate deep encoding on a neurophysiological level through the stronger synchronization of the same neuronal cell assemblies underlying conventional verbal learning and memory. In either case, the temporal structure implicit in musical stimuli may sharpen the timing of neural dynamics in brain networks degraded by demyelination in MS.

Based on Gestalt perception and learning principles, it has also been proposed (Janata et al., 2002; Wallace, 1994; Deutsch, 1982) that musical patterns engage effective mechanisms by incorporating temporal structure and redundancy that chunks information into more manageable units (Deutsch, 1999; Gfeller, 1983). “Chunking” is known to be not only a critical mechanism in declarative memory learning and recall, but also in motor learning (Verwey, 2001). Chunking as a mechanism in grouping and structuring perceptual information may be an innate feature across a broad phylogenetic range of nervous systems to optimize sensory acquisition (Matzel et al., 1988). The intrinsic structure of sound patterns in music is a highly effective mechanism to facilitate perceptual grouping and chunking. The present study supports the notion that “musical chunking” can be exploited to rehabilitate verbal learning and memory. It extends previous research regarding the beneficial effect of musical template learning on verbal learning in healthy adult subjects (Peterson and Thaut, 2003). It also extends findings from a previous pilot study, which suggested that music can improve memory in MS patients (Maeller, 1996), by also showing that music-induced enhancements were significantly correlated with increases in severity of disease. The present study is to our knowledge the first research to investigate physiological correlates of enhanced memory capability in MS using musical mnemonics. A physiological correlate of enhanced memory indicates that it is possible to induce plasticity in the neuronal network activity of the dysfunctional brain.

There is a growing body of knowledge about the neurobiological correlates of verbal memory, music, chunking, and plasticity. Lesion (Peretz, 2002; Halpern, 2001), functional imaging (Parsons et al., 1998; Smith et al., 1998; Platel et al., 1997; Zatorre et al., 1996), EEG (Peterson and Thaut, 2002; Ruchkin et al., 1997) and MEG (Maess et al., 2001; Tecchio et al., 2000; Makeig and Jung, 1996) studies have illustrated that both music and verbal memory recruit widespread networks encompassing many brain regions. Theoretical work is beginning to link the behavioral level phenomenon of chunking to the dynamics of cell assemblies in cortex (Wickelgren, 1999). System-level research on brain plasticity has highlighted the importance of temporal structure in stimuli (Pantev et al., 1999; Merzenich et al., 1993) and associated cortical plasticity with verbal learning (Tallal, 2000). Collectively, the research suggests that physiological measures of brain network dynamics (Garrett et al., 2003; Fuster, 1997), such as spectral analysis of the scalp EEG (Basar et al., 1999; Niedermeyer and Lopes da Silva, 1999), can provide a window into how musical chunking may enhance verbal memory.

In conclusion, we evaluated the use of musical mnemonics as a specific strategy for enhancing verbal memory in MS patients and to identify neural “markers” for short-term plasticity in brain network activity that correspond to learning and improved memory. The combination of behavioral and physiological measures is suggestive of the therapeutic potential and underlying neurobiological mechanisms of musical mnemonics in verbal learning and memory, an area of dysfunction in MS that is underserved in basic research and therapeutic intervention. The finding that music can improve word order memory is significant given the increasingly recognized cognitive deficits in MS (Amato et al., 2001; Rao, 1990; Peyser et al., 1980). This is the first known study to extend earlier work on memory for temporal order in MS (Beatty and Monson, 1991) using an ecologically salient paradigm like the RAVLT.

## Conflict of Interest Statement

The authors declare that the research was conducted in the absence of any commercial or financial relationships that could be construed as a potential conflict of interest.
